# 

**Published:** 1908-01-11

**Authors:** 


### CONTENTS.

| | PAGE |
|---|---|
| **A Discussion on the Complications of Pneumonia** | 381 |
| **The Physician in Art** ... ... ... ... ... ... | 382 |
| **Annotations—** | |
| The Church Bell Nuisance—Science and the Public—Medical Men and Death Certificates ... ... ... ... ... ... | 383 |
| **Medical Opinion and Movement** ... ... ... ... | 384 |
| **Hospital Clinics—** | |
| Diseases of the Tear Passages. By A. Maitland Ramsay, M.D., F.F.P.S. ... ... ... ... ... ... | 385 |
| Spinal Caries. By Donald Armour, B.A., M.B., F.R.C.S. ... | 389 |
| **Illustrative Cases—** | |
| Longitudinal and Other Sinus Thrombosis... ... ... ... | 391 |
| A Case of Red Granular Kidney ... ... ... ... ... | 392 |
| **Points in Surgery—** | |
| Fibro Adenomata of the Breast ... ... ... ... ... | 393 |
| Some Diseases of the Male Genital System... ... ... ... | 394 |
| **Diseases of Children—** | |
| Gastric Digestion in Babies ... ... ... ... ... ... | 395 |
| **Otology—** | |
| Concerning Operations for Otorrhœa... ... ... ... ... | 396 |
| **The General Practitioner's Column—** | |
| Some Points in the Treatment of Chorea ... ... ... ... | 397 |
| Practical Notes on Diagnosis and Treatment ... ... ... | 398 |
| **Therapeutics—** | |
| Prescriptions and Dispensing ... ... ... ... ... ... | 399 |
| **The Evolution of Medicine—** | |
| Old Masters of Medicine... ... ... ... ... ... ... | 400 |
| **Hospital Administration—** | |
| Current Hospital Topics ... ... ... ... ... ... ... | 401 |
| Liability of Operators and Anæsthetists ... ... ... ... | 402 |
| The Ramsey Cottage Hospital ... ... ... ... ... ... | 403 |
| King Edward's Hospital Fund for London... ... ... ... | 403 |
| News and Coming Events ... ... ... ... ... ... | 404 |
| The Graduates' Medical Schools ... ... ... ... ... | 404 |
| **Nursing Administration—** | |
| The Need for Trained Workhouse Matrons ... ... ... ... | 405 |
| The Common Task... ... ... ... ... ... ... ... | 406 |
| **Editor's Letter-Box—** | |
| Overworked Matrons: Educate the Committees... ... ... | 406 |

